# Security controls in an integrated Biobank to protect privacy in data sharing: rationale and study design

**DOI:** 10.1186/s12911-017-0494-5

**Published:** 2017-07-06

**Authors:** Takako Takai-Igarashi, Kengo Kinoshita, Masao Nagasaki, Soichi Ogishima, Naoki Nakamura, Sachiko Nagase, Satoshi Nagaie, Tomo Saito, Fuji Nagami, Naoko Minegishi, Yoichi Suzuki, Kichiya Suzuki, Hiroaki Hashizume, Shinichi Kuriyama, Atsushi Hozawa, Nobuo Yaegashi, Shigeo Kure, Gen Tamiya, Yoshio Kawaguchi, Hiroshi Tanaka, Masayuki Yamamoto

**Affiliations:** 1 Tohoku Medical Megabank Organization, Tohoku University, 2-1 Seiryo-machi, Aoba-ku, Sendai, Japan; 2 Graduate School of Medicine, Tohoku University, Sendai, Japan; 3 International Research Institute of Disaster Science, Tohoku University, Sendai, Japan; 4 Tohoku University Hospital, Tohoku University, Sendai, Japan; 5 Graduate School of Information Sciences, Tohoku University, Sendai, Japan

**Keywords:** Personalized healthcare, Biobank, Privacy violation risk, Personal genome data, Personal health data, Data sharing policy, Security policy, Remote access

## Abstract

**Background:**

With the goal of realizing genome-based personalized healthcare, we have developed a biobank that integrates personal health, genome, and omics data along with biospecimens donated by volunteers of 150,000. Such a large-scale of data integration involves obvious risks of privacy violation. The research use of personal genome and health information is a topic of global discussion with regard to the protection of privacy while promoting scientific advancement. The present paper reports on our plans, current attempts, and accomplishments in addressing security problems involved in data sharing to ensure donor privacy while promoting scientific advancement.

**Methods:**

Biospecimens and data have been collected in prospective cohort studies with the comprehensive agreement. The sample size of 150,000 participants was required for multiple researches including genome-wide screening of gene by environment interactions, haplotype phasing, and parametric linkage analysis.

**Results:**

We established the *T* *ohoku* *M* *edical* *M* *egabank* (*TMM) data sharing policy*: a privacy protection rule that requires physical, personnel, and technological safeguards against privacy violation regarding the use and sharing of data. The proposed policy refers to that of NCBI and that of the Sanger Institute. The proposed policy classifies shared data according to the strength of re-identification risks. Local committees organized by TMM evaluate re-identification risk and assign a security category to a dataset. Every dataset is stored in an assigned segment of a supercomputer in accordance with its security category. A security manager should be designated to handle all security problems at individual data use locations. The proposed policy requires closed networks and IP-VPN remote connections.

**Conclusion:**

The mission of the biobank is to distribute biological resources most productively. This mission motivated us to collect biospecimens and health data and simultaneously analyze genome/omics data in-house. The biobank also has the mission of improving the quality and quantity of the contents of the biobank. This motivated us to request users to share the results of their research as feedback to the biobank. The *TMM data sharing policy* has tackled every security problem originating with the missions. We believe our current implementation to be the best way to protect privacy in data sharing.

**Electronic supplementary material:**

The online version of this article (doi:10.1186/s12911-017-0494-5) contains supplementary material, which is available to authorized users.

## Background

The concept of personalized healthcare has brought about a paradigm shift from curing to prevention in medical activity. This concept proposes preventive medicine that takes individual genome and omics information into account [1]. The prospect of applying this concept has been dramatically improved by the recent development of genome and omics analyses, bioinformatics, and biobanks [2]. Biobanking was listed in “10 Ideas Changing the World Right Now” [3] and noted in the State of Union address by the U.S. President in 2015 [4]. Biobanks have promoted the development of drugs, the discovery of biomarkers, and the optimization of therapies, by providing information on stratified population by various kinds of genetic and environmental traits [2]. Biobanks have become indispensable to biomedical research communities studying personalized healthcare.

Recent progress in next-generation sequencing technologies has enabled speedy production of personal genome data [5]. Mass spectrometry and NMR spectrometry technologies have also been developed and have enabled high-throughput production of personal omics data, including proteomics and metabolomics. Such cutting-edge technologies facilitate large-scale analyses of personal genome and omics data with biospecimens stored in biobanks. Integration of genome/omics data and health data will greatly increase the value of biobanks, because the biobanks can support studies of genotype-phenotype correlations linking genetic variation with health outcomes [2]. We consider the analyses of genome and omics data using the donated biospecimens as an important task of biobanks. Biobanks have a mission of distributing biological resources most effectively and most productively. Unfortunately, biospecimens are doomed to finite resources and will be exhausted after a finite number of research uses. In contrast, genome and omics information can be used an infinite number of times; and information can be shared by an unlimited number of users any number of times. Ensuring such unlimited sharing of resources has greatly motivated us to establish a biobank, in which genome/omics data are analyzed and maintained in-house.

We have developed Tohoku Medical Megabank (TMM) biobank [6], which we refer to as an “integrated biobank”. The “integrated biobank” integrates personal health data and personal genome/omics data along with biospecimens donated by volunteers. Owing to this integration, the TMM biobank can provide biomedical research communities with not only biospecimens but also data derived from biospecimens. We collected biospecimens and health data, while simultaneously analyzing genome/omics data in-house, and integrated both datasets into the integrated biobank, so that these data could be shared with research communities to promote scientific advancement. Researchers are encouraged to donate their research results back to our biobank whenever possible. We believe that such donations or feedback from users greatly improve the quality and quantity of the integrated biobank. The goal is continued development and improvement of the integrated biobank, and we respectfully ask users to contribute to the goal. As of August 2016, the TMM biobank contains genome information on more than 10,000 volunteers [7], omics information on 1000 volunteers [8], and health information on more than 130,000 volunteers [6]. All of the volunteers are participants of prospective cohort studies.

Such a large-scale integration of health and genome/omics data involves privacy violation risks. A privacy protection standard on the use of personal health data was established by the U.S. government, which was called the Health Insurance Portability and Accountability Act (HIPAA) [9]. This act proposed protocols for mitigating privacy risks related to personal health data, and HIPAA and variations thereof have been adopted all over the world for use in a wide range of biomedical research. On the other hand, there is no global standard for privacy protection regarding the use of personal genome and omics data. Identifiability is evident with personal genome data [5, 10–12]; however, no appropriate way to mitigate the identifiability has yet been reported on the basis of methods such as suppression, generalization, and randomization that have been developed to mitigate the identifiability of personal health data disclosed [13]. Therefore, genome data should be protected rigorously through physical and technological security controls. Such a security control has been established by the NCBI in *NIH Security Best Practices for Controlled-Access Data* [18] and by the Sanger Institute in the *HUMAN GENETICS DATA SECURITY POLICY* [14]. We established a privacy protection rule for use and sharing of our genome data based on these policies. The proposed policy requires the implementation of physical, technical, and legal protection against privacy violations. The proposed policy also requires a contract with users to prohibit any attempts at re-identification. We believe that this is the best way available at present to protect an integrated biobank from privacy violation risks. While physical and technical guards protect violations from outsiders of integrated biobanks, both guards are powerless before violations from insiders involving staffs of biobanks and users of data sharing. If the staffs or the users intend to divulge confidential information, physical and technical guards are ineffective in protecting from such divulgences. In this regard, we surmise that legal contract can work effectively on such a security attack from the insiders and users [15]. Only a collective effort of physical, technical, and legal protection can be the best conceivable security policy.

The present paper reports a framework of privacy and security controls implemented in the integrated biobank. The proposed framework is composed of several security measures:

i. Define criteria for determining security risk of shared data,
ii. Designate a security manager at every research application,
iii. Separate genome data from health data in storage,
iv. Off-line data transfer at de-identification and anonymization,
v. Control every access to every security segment of the TMM supercomputer in closed networks,
vi. Control remote access to the closed networks,
vii. Informed consent that permits broad applications in research use,
viii. Legal agreements with users of the shared data.

The TMM biobank implements all the security measures, which produced innovative outputs such as:

- In-house analyses of genome/omics data, which greatly enhances quality and quantity of our shared data,
- Remote use of personal genome data by telecommunication, which greatly enhances availability of our data sharing.

Details are described in the following sections.

## Methods

### Outline of the TMM biobank

The mission of TMM is to facilitate the solution of medical problems in the aftermath of the Great East Japan Earthquake of March 11, 2011 (GEJE) by introducing personalized healthcare into the damaged areas. The TMM seeks to develop a biobank capable of integrating health and genome data from 150,000 volunteers [6]. Health data have been collected by the following two prospective cohort studies: TMM Community-Based Cohort Study that targets 80,000 adult volunteers, and TMM Birth and Three-Generation Cohort Study that targets70,000 volunteers consisting of infants and siblings, their parents, grandparents, and extended family members. Genome data are obtained by deep whole-genome DNA sequencing of donated blood or saliva [7]. Omics data are obtained by mass spectrometry and NMR spectrometry [8, 16]. In order to ensure the long-term usability of research data from these cohort studies, biospecimens and health and genome/omics data are archived in a biobank. The biospecimens and information are used and shared under a material transfer agreement (MTA), a data transfer agreement (DTA), and a collaborative research agreement (CRA), and only when the *Sample and Data Access Committee* approves a research application. Through these activities, we aim to realize personalized healthcare based on genetic and lifestyle information from the residents in the earthquake- and tsunami-damaged areas. These activities will be helpful in recovery efforts and in improving these residents’ health status. Our additional aim is to contribute to the restoration and development of the damaged areas by undertaking various research activities that utilize biospecimens and information in the TMM biobank [6].

The TMM project is jointly administered by Tohoku Medical Megabank Organization (ToMMo) at Tohoku University and Iwate Tohoku Medical Megabank Organization (IMM) at Iwate Medical University. While the prospective cohort studies are conducted by both the organizations, the TMM biobank is mainly maintained by the ToMMo, because the biobank is located at Tohoku University. The ToMMo takes the responsibility of securing the privacy of the data of the TMM biobank. The present paper describes the privacy protection framework constructed by the ToMMo. We are currently preparing another publication on the TMM biobank to address issues of collection, processing, and storage of biospecimens. The TMM biobank stores blood, urine, saliva, dental plaque, and breast milk donated from participants.

The inclusion criteria for the TMM Cohort Studies are: 1) persons aged 20 years or over; and 2) persons registered in the basic resident register of Miyagi Prefecture and Iwate Prefecture at the time of enrollment. For fetuses and children, inclusion criteria are: 1) fetuses of pregnant women who participated in the study; and 2) siblings of fetuses who participated in the study, regardless of kinship. In the TMM Community-Based Cohort Study, a sample size of 80,000 participants was required for genome-wide screening of gene by environment interactions with a minimum odds ratio ranging 1.4 to 29.1. In sample size calculation in the TMM Birth and Three-Generation Cohort Study, a sample size of approximately 70,000 participants from three generations was required for a multipurpose research platform, including haplotype phasing, parametric linkage analysis, cross-family identity by descent mapping, and family-based association studies such as the transmission-disequilibrium test and the corrected chi-squared test [6].

## Results

### TMM data sharing policy

**Fig. 1:**
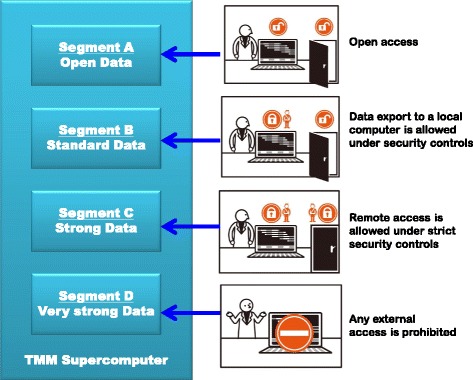
Segmented data storage in the TMM supercomputer. Segments A, B, C, and D are assigned to Open, Standard, Strong, and Very Strong data, respectively, in accordance with the security classification defined in the *TMM data sharing policy.* Segment A is open to the public and is freely accessible with no restriction. Segment B allows remote access and data export under the required security controls. Segment C allows remote access from security rooms equipped with all required security countermeasures. Segment D is rigorously closed and is prohibited from any remote access

We established *TMM data sharing policy* [17], since there were no rules in Japan for the use and sharing of personal health data including personal genome/omics data. This policy referred to *NIH Security Best Practices for Controlled-Access Data* [18] and the *HUMAN GENETICS DATA SECURITY POLICY* [14]. Although identifiability is evident with personal genome data [5, 10–12], no appropriate way to mitigate the identifiability has yet been reported. Therefore, we decided that data sharing should be protected by accurate evaluation of the re-identification risks and determination of physical and technological security controls. The *TMM data sharing policy* classifies shared data into four categories according to the strength of conceivable re-identification risks (Table 1 and Fig. 1):

**Table 1:** Security categories defined by the *TMM data sharing policy* and attributes of the categorized dataset

Security category^a^	Dataset^b^	Minimum necessary^c^	Identifiability^d^	Research use^e^	Remote access^f^
Very strong	Entire dataset	No	Significantly high	No	No
Strong	Extracted dataset	Yes	Significantly high	Yes	Yes, under security control, data transfer is not allowed
Standard	Extracted dataset	Yes	Negligible	Yes	Yes, under security control, data transfer is allowed
Open	Extracted dataset	No	No	Yes	Yes

^a^Security category defined by the *TMM data sharing policy*, ^b^Whole or part of sharable dataset in the TMM biobank, ^c^Applicability of the minimum necessary principle, ^d^Strength of identifiability, ^e^Allowance of research use, ^f^Availability of remote access

**Very strong:** An entire set of data in the TMM biobank consisting of an entire set of health data and an entire set of genome/omics data. All data are de-identified in compliance with the *HIPAA Limited Data Set* standard [19, 20]. The following section specifies which the *Limited Data Set* elements are included in the TMM shared data. Since we estimate the re-identification risk of these data to be significantly high, the data are strictly locked in a specific segment of the TMM supercomputer where any remote access is prohibited. Local access is only allowed through a thin-client terminal. Therefore, remote users must travel to the ToMMo headquarters located in Miyagi Prefecture to use data belonging to this category. Although browsing of data in this category is allowed under contract of the DTA, research using the data is not allowed because studies must be conducted with minimum necessary extractions from the entire dataset [9, 20] (i.e.*,* using Strong data described below).

**Strong:** A minimum necessary dataset extracted from the entire set of data in the TMM biobank (i.e.*,* Very strong data). A user can request retrieval of a minimum dataset for use in his/her research application. Research on data in this category is allowed following approval by the *Sample and Data Access Committee*. Since we estimate the re-identification risk of the data to be significantly high as with the Very strong data, the data are stored only in a specific segment of the TMM supercomputer. However, for the convenience of research applications, remote access is allowed only if all of the required security controls defined in the *TMM data sharing policy* are strictly followed. Technical details are provided in the “Network configuration” section of the present paper.

**Standard:** An extracted dataset with negligible re-identification risk in consideration of distinguishability and naming resource availability [21–23]. The *Sample and Data Access Committee* organized by the ToMMo and the IMM evaluates the re-identification risks of individual datasets belonging to this category. Data in this category can be exported to and used by remote computers provided that there is an approved research application under a DTA. The remote computer should be protected from any Internet access, and the security should be in strict compliance with the *TMM data sharing policy*.

**Open:** Data with no conceivable re-identification risk. Statistical data are included in this category. Data in this category can be disclosed to anyone without the need for a DTA. The data can also be exported without restriction and can be accessed through the Internet.

While Very strong and Strong data are stored securely in the TMM supercomputer, Standard and Open data are also stored in the TMM supercomputer. The TMM supercomputer serves as a platform for data use and sharing of different security categories. In order to control access to individual datasets belonging to different security categories, the computer storage is partitioned into four segments, one for each category (Fig. 1). Data transfer among the four segments is strictly controlled, and none of the segments is connected to any of the other segments. Data can only be transferred by a portable device equipped with data encryption and biometric authentication. All data transfer should be controlled by a security manager who is responsible for all security procedures involving data sharing. A principle investigator of an approved data use is obligated to designate a security manager to control data security in the research application.

The TMM has a special committee that evaluates re-identification risks of individual datasets and determines to which categories the datasets belong. To this end, the *Sample and Data Access Committee* has been established. This committee consists of nation-wide specialists from a wide variety of research fields, including human genetics, epidemiology, and jurisprudence, and the majority of the members and the chair do not belong to Tohoku University or Iwate Medical University which undertakes TMM projects. Tohoku University also organizes the *Committee for Data and Systems Integration* inside the organization for data monitoring, data cleaning, data quality control, and establishing and reviewing the data sharing policy and rules.

### Which the *Limited Data Set* elements are included in the TMM shared data

The TMM shared data includes two types of elements which are not listed as direct identifiers in the *HIPAA Limited Data Set* [19]:

1. **Elements of date:** Dates of data acquisition and sampling are included in the TMM shared data. Those dates are indispensable for the studies of time-dependent changes. The TMM shared data have the great advantage in time-dependent analysis in pathogenic progress, because the TMM biobank collects many kinds of time-dependent information with the changes in health conditions including pathogenesis. No other element of date is included in the TMM shared data.
2. **Geographic subdivisions:** Information on living in coastal or inland area is included. The mission of the TMM biobank is to facilitate the solution of medical problems in the aftermath of the Great East Japan Earthquake of March 11, 2011 [6]. Comparison of health data between coastal and inland area is indispensable for the investigation of damages caused by the Earthquake and Tsunami. No other geographic subdivision smaller than a state is included in the TMM shared data.

### Evaluation of re-identification risks of the TMM shared data

We evaluated re-identification risk on the basis of the model reported in [21]. This model proposed three measures of re-identification risks of a shared dataset: replication, resource availability, and distinguishability. Replication indicates the chances that the data will consistently occur. Resource availability indicates which external naming resource enables the re-identification and who is permitted to access to the external naming resource. Distinguishability indicates the extents which the data can be distinguished. Additional file 1: Table S1 summarizes our entire consideration in this section.

#### Strong and Very strong

We evaluate a dataset categorized as Strong or Very Strong to carry significantly high re-identification risk. The dataset includes 1) personal genome data and/or 2) medical histories of rare and incurable diseases. We evaluate the three measures of re-identification risks of 1) and 2) as follows:

**Replication:** Replication of both 1) and 2) is estimated to be high. Both 1) and 2) occur consistently for the entire lifetime.

**Resource Availability:** Resource availability of 1) differs from country to country. In Japan the availability is low, because there is no database service for retrieval of surnames using genetic variation data like Ysearch (www.ysearch.org) and SMGF [11]. None of Japanese surnames are provided by such a database service. However, resource availability of 2) is high, because a patient affected with such a disease is commonly identified by surface appearances or an action of everyday life of the patient.

**Distinguishability:** Distinguishability of both 1) and 2) is estimated to be high. Specifying DNA sequence at only 30 to 80 SNP positions will uniquely define a single person [5]. The TMM determined personal genome sequences of participants and discovered 21.2 million single-nucleotide variants in our assembled sequences [7]. Obviously distinguishability of the 1) is high. Distinguishability of 2) is also high because of the small number of the patients.

We conclude that re-identification risk of Strong or Very Strong data is significant high. Although resource availability of personal genome data is low in Japan at present, the re-identification risk should not be disregarded in consideration of the rapid advancement of technologies [5].

#### Standard

We evaluate a dataset categorized as Standard to carry negligible re-identification risk with the same model as the one used in evaluation of Strong or Very Strong datasets [21], in which we do not adopt any numeric thresholds. Datasets in Standard category must not include personal genome data nor medical histories of rare and incurable diseases, both of which are categorized into Strong or Very Strong. Then the Standard datasets include 1) demographics and 2) laboratory and physiological measurements, and questions on sociodemographic factors and lifestyle habits, and medical histories of common diseases. We evaluate the three measures of re-identification risks of 1) and 2) as follows:

**Replication:** In general, replication of 1) is high; however, replication of 2) is regarded as low [21, 24].

**Resource availability:** Naming resources are not available for 2) in general. However, naming resources are available for 1); a voter registration list has been a well-studied example of the naming resources [13, 21, 23, 25]. Availability of voter registration lists depends on the legal systems of local administrative districts like cities, prefectures, and states. In USA, a voter registration list can be purchased as an electronic file, which has been demonstrated to actually cause the re-identification. In Japan, voter registration lists are available on request in free. Names, addresses, and birthdates of voters are disclosed. Only reading books or transcribing from books is allowed, so that obtaining electronic data is impossible. The books are archived in local election administration offices disseminated at more than 1700 local districts all over Japan [26]. Therefore, compiling a comprehensive electronic list of voters is extremely difficult in Japan.

**Distinguishability:** Distinguishability of 1) is estimated as low. The TMM shared data include only three demographics elements: gender, age, and separation of coastal and inland about living areas. K-anonymity of the three demographics elements does not exceed k = 10 threshold. Distinguishability of 2) is estimated as high. TMM cohort studies collect 1099 attributes of laboratory and physiological measurements, and 3070 attributes of questions on sociodemographic factors and lifestyle habits, and medical histories of common diseases. Because of such a large variety of collected attributes, distinguishability of the data becomes naturally high.

We concluded that re-identification risk of Standard datasets is negligible. Privacy violation could occur when all the three measures (replication, resource availability, and distinguishability) reach high levels all at once [21], which is not the case of the datasets of Standard category.

#### Open

We evaluate a dataset categorized as Open to carry no conceivable re-identification risk. The dataset includes only summary level data and never includes participant/subject-level data. To be precise, the dataset consists of statistic scores on the overall dataset.

### Data management

The TMM biobank involves the in-house analysis of genome and omics data, which is a distinctive characteristic of the biobank. In general, biobanks turn over the analyses of genome/omics data to third parties or outside institutes. For example, UK biobank [27] turns over that to the Sanger Institute, and BioVU [28] turns over that to commercial third parties. In-house analyses of genome/omics data have the advantage of continuous biobanking operation. In the TMM biobank, both biospecimens and genome/omics data are managed by the same IDs, which are referred to as “Biobank IDs”. The continuity of ID management allows genome and omics analyses to be performed more simply and speedier. We can easily check errors and unexpected incidents that occurred in genome and omics analyses by looking up information stored in the biobank without ID conversion. Moreover, we can speed up both the forwarding of samples to genome and omics teams and the feeding back of results to the biobank.

In a discussion of ethical and legal considerations on the use, storage, and sharing of genome data [29–32], there have been recommendations of completely separate storage of genome data in order to protect against the high potential for confidentiality breach risks [33–35]. Others have argued that genome data are not sufficiently different from other health data to justify different security measures [31, 34–36]. This is a controversial issue, and individual institutes have to decide their policies independently. We decided that biospecimens and genome/omics data are managed by the same IDs, i.e.*,* Biobank IDs, while health data are managed by QA_test IDs, in completely separate storage from genome/omics data.

**Fig. 2:**
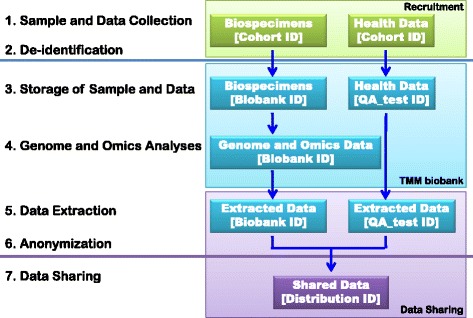
Data processing operations. Biospecimens and health data are collected with Cohort IDs. The De-identification process converts Cohort IDs into Biobank IDs in the case of biospecimens, while the process converts Cohort IDs into QA_test IDs in the case of health data. The TMM biobank stores biospecimens with Biobank IDs and health data with QA_test IDs. Genome and omics data are analyzed and stored with Biobank IDs. After the approval of a research application, datasets of minimum necessary data are extracted from the TMM biobank. The extracted datasets are combined and anonymized by ID conversion into Distribution IDs. The combined dataset is then used and shared with Distribution IDs for the approved research application

The data are managed by four types of IDs in data processing operations (Fig. 2):

**Cohort ID:** IDs used with biospecimens and health data before de-identification. Cohort IDs are in personal identifiers, because volunteers’ names and addresses are directory linked to Cohort IDs. Cohort IDs are used to contact volunteers and collect biospecimens and health data.

**Biobank ID:** IDs used with biospecimens and genome/omics data after de-identification. Biobank IDs have no link to any personal identifier. Biobank IDs are used for the storage of biospecimens and for analyses and storage of gnome/omics data.

**QA_test ID:** IDs used with health data after de-identification. QA_test IDs have no link to any personal identifier. QA_test IDs are used for the storage of health data. Thus, health data are stored separately from genome/omics data for security reasons.

**Distribution ID:** IDs used for the use and sharing of data. After approval of data sharing, two types of datasets are extracted from the TMM biobank: a dataset of genome/omics data with Biobank IDs and a dataset of heath data with QA_test IDs. Both datasets are combined and anonymized by ID conversion into Distribution IDs. Distribution IDs are assigned differently on a user-by-user basis in order to prevent matching the data between the two parties who share the data. Distribution IDs are used at research applications of the combined data of genome/omics and health.

### De-identification and anonymization

Since personal identifiability is intrinsic to health and genome/omics data, the identifiability should be mitigated appropriately prior to any research use of the data. In our data processing operations, two mitigation steps protect privacy violation risks: de-identification and anonymization. Biospecimens are de-identified in advance of genome/omics analyses in compliance with *Ethical Guidelines for Human Genome/Gene Analysis Research* (Japanese Government) [37]. Health and genome/omics data is anonymized in advance of data sharing in accordance with the *TMM data sharing policy* [17]. The de-identification and anonymization protocols are designed as follows (Fig. 2):

**De-identification:** Direct identifiers are removed, but indirect identifiers such as ages, residential areas (coastal or inland), and registration and examination dates remain intact, in accordance with the *HIPAA Limited Data Set* standard [19]. IDs are converted from Cohort IDs to Biobank/QA_test IDs. The data preserve linkages to direct identifiers, which could only be re-linked by a trusted third party. A designated person maintains the linkages and manages the access to the linkages for de-identification. The person should not be engaged in the TMM project and be in a professional position with a duty of confidentiality.

**Anonymization:** IDs are converted from Biobank/QA_test IDs to Distribution IDs. Data are irretrievably unlinked from direct identifiers. The anonymized datasets are used in research applications. The *Sample and Data Access Committee* assigns security categories to individual datasets and determine the security controls required for research uses in accordance with the *TMM data sharing policy*. Please note that, in the present paper, anonymization does not imply removal of re-identification risks, but rather implies evaluation of re-identification risks and provision of security countermeasures in accordance with the security category.

Since we request users to share the results of their research as feedback to the TMM biobank, we preserve ID conversion tables between Biobank/QA_test IDs and Distribution IDs. As such, the anonymization is regarded as linked-anonymization inside of the TMM biobank. On the other hand, the anonymization is regarded as unlinked-anonymization outside of the TMM biobank, i.e.*,* at the place of use and sharing of data. This is because any ID convertible relation is irretrievably unlinked at data sharing. The ID conversion table is kept under lock and key inside of the TMM. We do not employ any particular algorithm (i.e. One-way hashing) to produce the table, but no relation in the table overlaps each other.

### TMM supercomputer

The TMM supercomputer is a pivotal piece of infrastructure for the genome cohort studies and plays a central role in the storage and analysis of genome/omics and health data. The TMM supercomputer consists of 16,000 CPU cores, 111 TB of memory, 18 PB of disk storage, and a 56-Gbps network [38].

### Network configuration

**Fig. 3:**
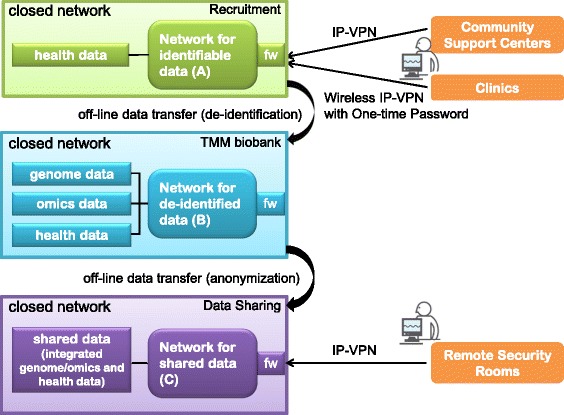
Network configuration. The network configuration consists of three types of closed sub-networks; A) a network for identifiable data, B) a network for de-identified data, and C) a network for shared data. Only de-identification or anonymization actions can traverse between network A and network B or network B and network C, respectively. The two actions require off-line data transfers using portable devices with biometric authentication. VPN connections enable remote access from distant places of recruitment (community support centers and clinics) and data sharing (remote security rooms). An IP-VPN or wireless IP-VPN with a one-time password system meets the technological requirements of the security policy. The TMM biobank does not allow any remote access

Networks are configured so as to ensure the most secure data management and data transference in the TMM biobank. The network configuration consists of three sub-networks (Fig. 3): A) a network in which personal identifiable data are transferred, B) a network in which de-identified data of genome, omics, and health are transferred, and C) a network in which data of use and sharing are transferred. Three sub-networks are independent and are controlled by independent firewalls. Moreover, the networks are not interconnected and are not connected to the Internet. Only de-identification or anonymization actions can traverse the disconnections between the closed sub-networks. The traversing actions are controlled off-line, and portable devices with biometric authentication should be used for data transport in the traversing actions.

All of the sub-networks are closed networks. However, the networks should allow remote access. The sub-network for personal identifiable data should be accessed from places where we contact volunteers (Fig. 3(A)). Volunteers are recruited at Community Support Centers located in seven districts in Miyagi Prefecture, and at over 50 obstetrics and gynecology clinics located all over Miyagi Prefecture [6]. Our recruitment staffs interview volunteers and receive information consent, biospecimens, and health data from the volunteers at the Community Support Centers and the obstetrics and gynecology clinics. All of the recruitment places are distant from the ToMMo headquarters. We then constructed Internet Protocol Virtual Private Network (IP-VPN) connections between the ToMMo headquarters and the Community Support Centers. The IP-VPN connections were possible because all of the Community Support Centers are owned by the ToMMo.

On the other hand, IP-VPN connections to the over 50 clinics cost a lot and burden us with technical problems. Construction of IP-VPN requires engineering works at every clinic to modify its network configurations. Most of the clinics were disinclined for the engineering works in behalf of ToMMo’s recruitments. Thus we decided to use an IP-VPN over a fourth-generation mobile telecommunications network called wireless IP-VPN, to connect portable PCs and the ToMMo headquarters. The recruiting staffs carry portable PCs into individual obstetrics and gynecology clinics and send health data through the wireless IP-VPN connections. Since the wireless IP-VPN has a security defect in that it can be abused if a portable PCs were to be stolen, we implemented the following security countermeasures: a one-time password log-in system involving security tokens, a virtual desk-top system that makes a portable PC behave as a thin client, and a device control system that prohibits the use of removable devices.

Remote access is also required for data sharing. In principle, data must be shared and used only in the TMM supercomputer. Users must travel to the ToMMo headquarters in Miyagi Prefecture, where the supercomputer is located. However, a remote access from a security room is allowed if and only if a user equips the room with all security countermeasures required by the *TMM data sharing policy*. The policy requires the following physical, personnel, and technical safeguards: an IP-VPN should connect the remote security room and the TMM supercomputer. The remote security room should be equipped with entrance-control devices with biometric authentication accompanied by surveillance cameras at all entrances. The remote access terminal should be a thin client that has no hard drive. The remote access user should enter into a contract with the ToMMo stating that they will make no attempt at re-identification. Access logs should be reviewed by the ToMMo headquarters on a regular basis. We have launched five remote security rooms, and several additional launches are under preparation.

We constructed thin client systems that control all the accesses from different locations with distinct security guards into right destinations of data storages. Any wrong access is rigorously blocked by the thin client systems. Additional file 2: Figure S1 illustrates the logical network configuration including the access controls by the thin client systems on the basis of the *TMM data sharing policy*. The networks have run over 4 years and nearly 500 users in total daily use the networks. The statistics of working status of the networks is shown in Additional file 3: Table S2.

### Informed consent with permission to share genome and omics data

TMM biobank serves as a data repository for a wide variety of research applications. When donations of biospecimens and data are provided through the comprehensive agreement method referred as “broad consent” [39], the donated biospecimens and data can be used in any research application without prior specification at the time of donation with a later opt-out for individual research uses and a guarantee of withdrawal. The informed consent also includes the volunteers’ permission to analyze genome and omics data using biospecimens. In their consent, the volunteers permit the TMM to maintain their genome and omics data in databases and to share their genome and omics data with academic and industrial research institutes outside of the TMM after approval by the *Sample and Data Access Committee*. Data sharing, in the case of genome data in particular, strictly requires the written consent of a donor in advance of sampling [29, 40]. The informed consent of the TMM biobank complies with this requirement.

Our informed consent does not define how the data will be used after the collection; however, the informed consent precisely defines what kind of specimens and data and how much volume of specimens the volunteers will donate to us. Therefore, only the use of specimens and data is “broad”. If a research application will collect additional specimens and/or data from the participants beyond the predetermined kinds and amounts, the participants will be asked to agree with a new informed consent that will clearly state the additional collections.

### Data transfer agreement and collaborative research agreement

The *TMM data sharing policy* requires a user to enter into a contract with the ToMMo for sharing data belonging to the Standard, Strong, or Very strong category. The contract consists of a DTA and a CRA. The DTA and the CRA play important roles in protecting privacy by restricting user activities relating to confidentiality breach risks. Four types of user activities are restricted by the DTA and the CRA:

A. Prohibition of data use for any purposes not included in the DTA or the CRA.
B. Prohibition of data use by any user not included in the DTA or the CRA.
C. Prohibition of attempts at re-identification of volunteers or any contact with volunteers, wherein volunteers are individuals who donate biospecimens and health data to the TMM biobank.
D. Prohibition of provision of re-edited data to any third parties without approval by the ToMMo.

We cancel the agreement immediately after any of the provisions is violated. We demand the violator to completely delete all the data shared by the TMM and the data derived from the shared data.

## Discussion

The use of personal genome information is a topic of global discussion with regard to the protection of privacy while promoting scientific advancement [30–32, 41]. Issues include the need for strong protection against privacy violations, appropriate informed consent, and maximizing public benefit [29]. Security policies for the sharing of personal genome data; e.g.*, NIH Security Best Practices for Controlled-Access Data* [18] by the NCBI and the *HUMAN GENETICS DATA SECURITY POLICY* [14] by the Sanger Institute have been proposed. Both of these policies outline physical and technological safeguards against data divulgences. These policies have been implemented in eMERGE [32, 40], the NBDC Human Database [42], and the TMM biobank. The present paper describes our challenges of applying these security policies to the integrated biobank, considering the in-house analysis of genome/omics data and the request for donating the results of research conducted by users. There is an increasing demand for a highly secure platform where personal genome data are collected, analyzed, and shared in association with personal health data [43–45]. The TMM biobank demonstrates a concrete example of the way such a highly secure platform is operated and actively used.

Even though security countermeasures are implemented, sensitive information could be divulged through data sharing. A novel technology encrypts all data in every computational process so that any data divulged will not be useful without first being deciphered [15]. This technology is called privacy-preserving analysis, and its application to genome-wide association study (GWAS) has been vigorously studied and applied. Privacy-preserving GWAS analysis enables the discovery of statistically significant correlations between a certain phenotype and genotype without disclosing genotype and phenotype data [46–50]. Although this is a promising technology, such a technology involves a weakness in that it can be applied only to a pre-determined analysis, which, in this case, is GWAS. In consideration of this weakness, we intend to apply privacy-preserving analysis technology to certain routine analyses of the TMM biobank in the future.

Beside health data, specificity of genome data results from certain essential features: 1) an association with traits and certain diseases, 2) identification capabilities, and 3) revelation of family relations [15, 30, 43–45, 51]. This paper describes our efforts to protect against “identification capabilities” of the personal genomes. In the TMM biobank, the personal genomes have not yet been interpreted their “associations with traits and certain diseases”, although the personal genomes have been genotyped and resulted in the discovery of thousands of variations [7]. In our next step, we intend to interpret all the genetic variations medically and to return the individual disease risks to the participants on the basis of the interpretations. In general, the medical interpretation is difficult to be completed by a single party; and a multiparty computational process can be vulnerable to security attacks [15]. We should develop new security protocols that protect the multiparty interpretation processes as well as the medically interpreted personal genomes in the foreseeable future.

On the other hand, “revelation of family relation” imposes no major risk in Japan. This is because there is no database service for retrieval of surnames and pedigrees associated with personal genetic variations of Japanese population, which enables inference of surnames from shared genome information [11]. In Japan, only a member of a pedigree is allowed to obtain his or her pedigree information from local governments, therefore there is no chance for systematical compilation of family pedigrees information [52].

## Conclusion

Biobanks have facilitated the sharing of large-scale data include personal genome data. Identifiability is evident with personal genome data, and there has been no report on an appropriate way to mitigate the identifiability. Therefore, genome data should be protected rigorously through physical and technological security safeguards. We established the *TMM data sharing policy* on the basis of precedent security policies put forth by the NCBI (http://law.e-gov.go.jp/htmldata/S22/S22HO224.html) and the Sanger Institute [14]. This is our challenge to apply the precedent policies to our special interest in analyzing genome/omics data in-houses at development of the biobank as well as requesting users to share the results of their research as feedback to the biobank. Our plans, current attempts, and accomplishments will be reviewed internally and externally, and the results will be reported elsewhere.

## Additional files

Additional file 1: Table S1. Evaluation of re-identification risks of the TMM shared datasets. This table summarizes how we evaluate re-identification risks of the TMM shared datasets categorized into Very Strong, Strong, Standard, and Open. (DOCX 15 kb)

Additional file 2: Figure S1. Logical configuration of the networks that form an infrastructure of the TMM biobank. Configuration of the TMM network is specified. (ZIP 53 kb)

Additional file 3: Table S2. Working status of networks in the TMM biobank. The statistics of working status of the TMM networks is shown. The network configuration is shown in Additional file 2: Figure S1. (DOCX 12 kb)

## Abbreviations

CRA: Collaborative Research Agreement
DTA: Data Transfer Agreement
GWAS: Genome-wide Association Study
HIPAA: Health Insurance Portability and Accountability Act
IMM: Iwate Tohoku Medical Megabank Organization
IP-VPN: Internet Protocol Virtual Private Network
MTA: Material Transfer Agreement
NCBI: National Center for Biotechnology Information
NIH: National Institutes of Health
TMM: Tohoku Medical Megabank
ToMMo: Tohoku Medical Megabank Organization

## References

[1] Collins FS, Varmus H (2015). A new initiative on precision medicine. N Engl J Med.

[2] Kinkorova J (2015). Biobanks in the era of personalized medicine: objectives, challenges, and innovation: overview. EPMA J.

[3] Parks A. 10 ideas of changing the world right now. Time. 2009:173(11).

[4] Remarks by the President in State of the Union Address | January 20, 2015 [https://www.whitehouse.gov/the-press-office/2015/01/20/remarks-president-state-union-address-january-20-2015]. Accessed 1 July 2017.

[5] Lin Z, Owen AB, Altman RB (2004). Genetics. Genomic research and human subject privacy. Science.

[6] Kuriyama S, Yaegashi N, Nagami F, Arai T, Kawaguchi Y, Osumi N, Sakaida M, Suzuki Y, Nakayama K, Hashizume H (2016). The Tohoku medical megabank project: design and mission. J Epidemiol.

[7] Nagasaki M, Yasuda J, Katsuoka F, Nariai N, Kojima K, Kawai Y, Yamaguchi-Kabata Y, Yokozawa J, Danjoh I, Saito S (2015). Rare variant discovery by deep whole-genome sequencing of 1,070 Japanese individuals. Nat Commun.

[8] Koshiba S, Motoike I, Kojima K, Hasegawa T, Shirota M, Saito T, Saigusa D, Danjoh I, Katsuoka F, Ogishima S (2016). The structural origin of metabolic quantitative diversity. Sci Rep.

[9] HIPAA_Privacy_Rule: MINIMUM NECESSARY. Standards for privacy of individually identifiable health information, Final Rule 2002, 45 CFR 164.502(b), 164.514(d).

[10] Homer N, Szelinger S, Redman M, Duggan D, Tembe W, Muehling J, Pearson JV, Stephan DA, Nelson SF, Craig DW (2008). Resolving individuals contributing trace amounts of DNA to highly complex mixtures using high-density SNP genotyping microarrays. PLoS Genet.

[11] Gymrek M, McGuire AL, Golan D, Halperin E, Erlich Y (2013). Identifying personal genomes by surname inference. Science.

[12] Malin B, Sweeney L. Determining the identifiability of DNA database entries. Proc AMIA Symp. 2000:537–41. PMC2244110 11079941

[13] Malin B, Benitez K, Masys D (2011). Never too old for anonymity: a statistical standard for demographic data sharing via the HIPAA privacy rule. J Am Med Inform Assoc.

[14] HUMAN GENETICS DATA SECURITY POLICY [http://www.sanger.ac.uk/legal/assets/data_sharing_policy_guidelines-june2016_v9-5.pdf]. Accessed 1 July 2017.

[15] Naveed M, Ayday E, Clayton EW, Fellay J, Gunter CA, Hubaux JP, Malin BA, Wang X. Privacy in the Genomic Era. ACM Comput Surv. 2015;48(1):6. 10.1145/2767007 PMC4666540 26640318

[16] Saigusa D, Okamura Y, Motoike IN, Katoh Y, Kurosawa Y, Saijyo R, Koshiba S, Yasuda J, Motohashi H, Sugawara J (2016). Establishment of protocols for global Metabolomics by LC-MS for biomarker discovery. PLoS One.

[17] TMM data sharing policy [http://www.megabank.tohoku.ac.jp/english/sample/]. Accessed 1 July 2017.

[18] NIH Security Best Practices for Controlled-Access Data [www.ncbi.nlm.nih.gov/projects/gap/pdf/dbgap_2b_security_procedures.pdf]. Accessed 1 July 2017.

[19] HIPAA_Privacy_Rule. RESEARCH. Standards for privacy of individually identifiable health information, Final Rule 2002, 45 CFR 164.501, 164.508, 164.512(i) [https://www.hhs.gov/hipaa/for-professionals/special-topics/research/index.html]. Accessed 1 July 2017.

[20] Sengupta S, Calman NS, Hripcsak G (2008). A model for expanded public health reporting in the context of HIPAA. J Am Med Inform Assoc.

[21] Malin B, Loukides G, Benitez K, Clayton EW (2011). Identifiability in biobanks: models, measures, and mitigation strategies. Hum Genet.

[22] Jiang X, Sarwate AD, Ohno-Machado L (2013). Privacy technology to support data sharing for comparative effectiveness research: a systematic review. Med Care.

[23] Sweeney L (1998). Privacy and medical-records research. N Engl J Med.

[24] Atreya RV, Smith JC, McCoy AB, Malin B, Miller RA (2013). Reducing patient re-identification risk for laboratory results within research datasets. J Am Med Inform Assoc.

[25] Malin B, Karp D, Scheuermann RH (2010). Technical and policy approaches to balancing patient privacy and data sharing in clinical and translational research. J Investig Med.

[26] List_of_cities_in_Japan [https://en.wikipedia.org/wiki/List_of_cities_in_Japan]. Accessed 1 July 2017.

[27] Elliott P, Peakman TC, Biobank UK (2008). The UK Biobank sample handling and storage protocol for the collection, processing and archiving of human blood and urine. Int J Epidemiol.

[28] Roden DM, Pulley JM, Basford MA, Bernard GR, Clayton EW, Balser JR, Masys DR (2008). Development of a large-scale de-identified DNA biobank to enable personalized medicine. Clin Pharmacol Ther.

[29] Shoenbill K, Fost N, Tachinardi U, Mendonca EA (2014). Genetic data and electronic health records: a discussion of ethical, logistical and technological considerations. J Am Med Inform Assoc.

[30] Nishimura AA, Tarczy-Hornoch P, Shirts BH (2014). Pragmatic and ethical challenges of incorporating the genome into the electronic medical record. Curr Genet Med Rep.

[31] McGuire AL, Fisher R, Cusenza P, Hudson K, Rothstein MA, McGraw D, Matteson S, Glaser J, Henley DE (2008). Confidentiality, privacy, and security of genetic and genomic test information in electronic health records: points to consider. Genet Med.

[32] Clayton EW, Smith M, Fullerton SM, Burke W, McCarty CA, Koenig BA, McGuire AL, Beskow LM, Dressler L, Lemke AA (2010). Confronting real time ethical, legal, and social issues in the electronic medical records and genomics (eMERGE) consortium. Genet Med.

[33] Green MJ, Botkin JR (2003). “genetic exceptionalism” in medicine: clarifying the differences between genetic and nongenetic tests. Ann Intern Med.

[34] Ross LF (2001). Genetic exceptionalism vs. paradigm shift: lessons from HIV. J Law Med Ethics.

[35] Rothstein MA (2007). Genetic exceptionalism and legislative pragmatism. J Law Med Ethics.

[36] Burke W, Pinsky LE, Press NA (2001). Categorizing genetic tests to identify their ethical, legal, and social implications. Am J Med Genet.

[37] Ethical Guidelines for Human Genome/Gene Analysis Research [http://www.lifescience.mext.go.jp/files/pdf/n796_00.pdf]. Accessed 1 July 2017.

[38] ToMMo Super Computer [https://sc.megabank.tohoku.ac.jp/en/outline]. Accessed 1 July 2017.

[39] Wendler D (2012). Consent for research with biological samples: one-time general consent versus a gift model. Ann Intern Med.

[40] McGuire AL, Basford M, Dressler LG, Fullerton SM, Koenig BA, Li R, McCarty CA, Ramos E, Smith ME, Somkin CP (2011). Ethical and practical challenges of sharing data from genome-wide association studies: the eMERGE consortium experience. Genome Res.

[41] Privacy and Progress in Whole Genome Sequencing, [http://bioethics.gov/sites/default/files/PrivacyProgress508_1.pdf]. Accessed 1 July 2017.

[42] NBDC Security Guidelines for Human Data (for Data Users) ver. 2.0 [http://humandbs.biosciencedbc.jp/en/guidelines/security-guidelines-for-users]. Accessed 1 July 2017.

[43] Hafen E, Kossmann D, Brand A (2014). Health data cooperatives - citizen empowerment. Methods Inf Med.

[44] den Dunnen JT (2015). The DNA Bank: high-security Bank accounts to protect and share your genetic identity. Hum Mutat.

[45] Ball MP, Thakuria JV, Zaranek AW, Clegg T, Rosenbaum AM, Wu X, Angrist M, Bhak J, Bobe J, Callow MJ (2012). A public resource facilitating clinical use of genomes. Proc Natl Acad Sci U S A.

[46] Zhang Y, Dai W, Jiang X, Xiong H, Wang S (2015). FORESEE: fully outsourced secuRe gEnome study basEd on homomorphic encryption. BMC Med Inform Decis Mak.

[47] Zhang Y, Blanton M, Almashaqbeh G (2015). Secure distributed genome analysis for GWAS and sequence comparison computation. BMC Med Inform Decis Mak.

[48] Lu WJ, Yamada Y, Sakuma J (2015). Privacy-preserving genome-wide association studies on cloud environment using fully homomorphic encryption. BMC Med Inform Decis Mak.

[49] Kim M, Lauter K (2015). Private genome analysis through homomorphic encryption. BMC Med Inform Decis Mak.

[50] Constable SD, Tang Y, Wang S, Jiang X, Chapin S (2015). Privacy-preserving GWAS analysis on federated genomic datasets. BMC Med Inform Decis Mak.

[51] Kostkova P, Brewer H, de Lusignan S, Fottrell E, Goldacre B, Hart G, Koczan P, Knight P, Marsolier C, McKendry RA (2016). Who owns the data? Open data for healthcare. Front Public Health.

[52] Family Register Act, Japan. [http://law.e-gov.go.jp/htmldata/S22/S22HO224.html]. Accessed 1 July 2017.

