# Supplementary material for: Security controls in an integrated Biobank to protect privacy in data sharing: rationale and study design
Source: BMC Med Inform Decis Mak. 2017 Jul 6;17:100. doi: 10.1186/s12911-017-0494-5 (PMC5501115; doi:10.1186/s12911-017-0494-5)
Supplement: Supplementary file 1 — Evaluation of re-identification risks of the TMM shared datasets. This table summarizes how we evaluate re-identification risks of the TMM shared datasets categorized into Very Strong, Strong, Standard, and Open. (DOCX 15 kb) [file 12911_2017_494_MOESM1_ESM.docx]

**Additional file 1: Table S1: Evaluation of re-identification risks of the TMM shared datasets.**

| **Security classification ^1^** | **Re-identification risk^2^** | **Included data^3^** | **Excluded data^4^** | **Replication^5^** | **Resource availability^6^** | **Distinguishability^7^** |
| --- | --- | --- | --- | --- | --- | --- |
| Very Strong, Strong | Significantly high | Personal genome data | None | High | Low in Japan | High |
|  |  | Medical histories of rare diseases | None | High | High | High |
| Standard | Negligible | Demographics | Personal genome data and medical histories of rare diseases | High | Low in Japan | Low |
|  |  | Laboratory and physiological measurements, questions on sociodemographic factors and lifestyle habits, and medical histories of common diseases |  | Low | Low | High |
| Open | No conceivable risk | Statistic scores in summary level | Participant/subject-level data | Low | Low | Low |

^1^Security classification defined by the *TMM data sharing policy*, ^2^Re-identification risk evaluated in the *TMM data sharing policy*, ^3^Inclusion data type, ^4^Exclusion data type, ^5^The level of replication, ^6^The level of resource availability, ^7^The level of distinguishability.
