# Supplementary material for: Security controls in an integrated Biobank to protect privacy in data sharing: rationale and study design
Source: BMC Med Inform Decis Mak. 2017 Jul 6;17:100. doi: 10.1186/s12911-017-0494-5 (PMC5501115; doi:10.1186/s12911-017-0494-5)
Supplement: Supplementary file 2 — Logical configuration of the networks that form an infrastructure of the TMM biobank. Configuration of the TMM network is specified. (ZIP 53 kb) [file 12911_2017_494_MOESM2_ESM.zip › Supplementary_DocumentR3_1.docx]

**Supplementary Document 3**

**Legends of Supplementary Figure 1**

**Supplementary Figure 1**: **Logical configuration of the networks that form an infrastructure of the TMM biobank.**

Logical network configuration for our secured data collection, storage, and sharing is illustrated. As shown in Figure 3, any access from the Internet is processed by one of three firewalls (FWs): FW-A for identifiable data, FW-B for de-identified data, and FW-C for shared data. Virtual Local Area Network (VLAN) under FW-A can be connected both from physical PCs and virtual PCs, while the other VLANs can be connected only from virtual PCs. Thin client system 1, 2, and 3 control all the connections from the virtual PCs to the individual VLANs where data are separately stored depending on the assigned security categories defined in the ToMMo data sharing policy. The thin client systems take charge of access controls from different locations with different security guards into right destinations of the data storages. Any wrong access is rigorously blocked by the thin client systems. Wired connections from clients should be authenticated by biometric authentication. PCs in the ToMMo headquarters and PCs in the remote security rooms, both of which are used in the analyses of personal genome and health data, are connected with biometric authentication. On the other hand, wireless connections from clients should be authenticated by one-time password. Portable PCs used by our recruiting staffs in obstetrics and gynecology clinics are connected with one-time password authentication.

**List of abbreviations in Supplementary Figure 1:**

4G, 4th generation; FW, firewall; IP-VPN, Internet Protocol-Virtual Private Network; LAN, Local Area Network; PC, Personal Computer; ToMMo, Tohoku Medical Megabank Organization; VLAN, Virtual Local Area Network; VRF, Virtual Routing Forwarding.
