# Supplementary material for: Security controls in an integrated Biobank to protect privacy in data sharing: rationale and study design
Source: BMC Med Inform Decis Mak. 2017 Jul 6;17:100. doi: 10.1186/s12911-017-0494-5 (PMC5501115; doi:10.1186/s12911-017-0494-5)
Supplement: Supplementary file 2 — Logical configuration of the networks that form an infrastructure of the TMM biobank. Configuration of the TMM network is specified. (ZIP 53 kb) [file 12911_2017_494_MOESM2_ESM.zip › Supplementary_Figure1R3.pptx]

## Slide 1
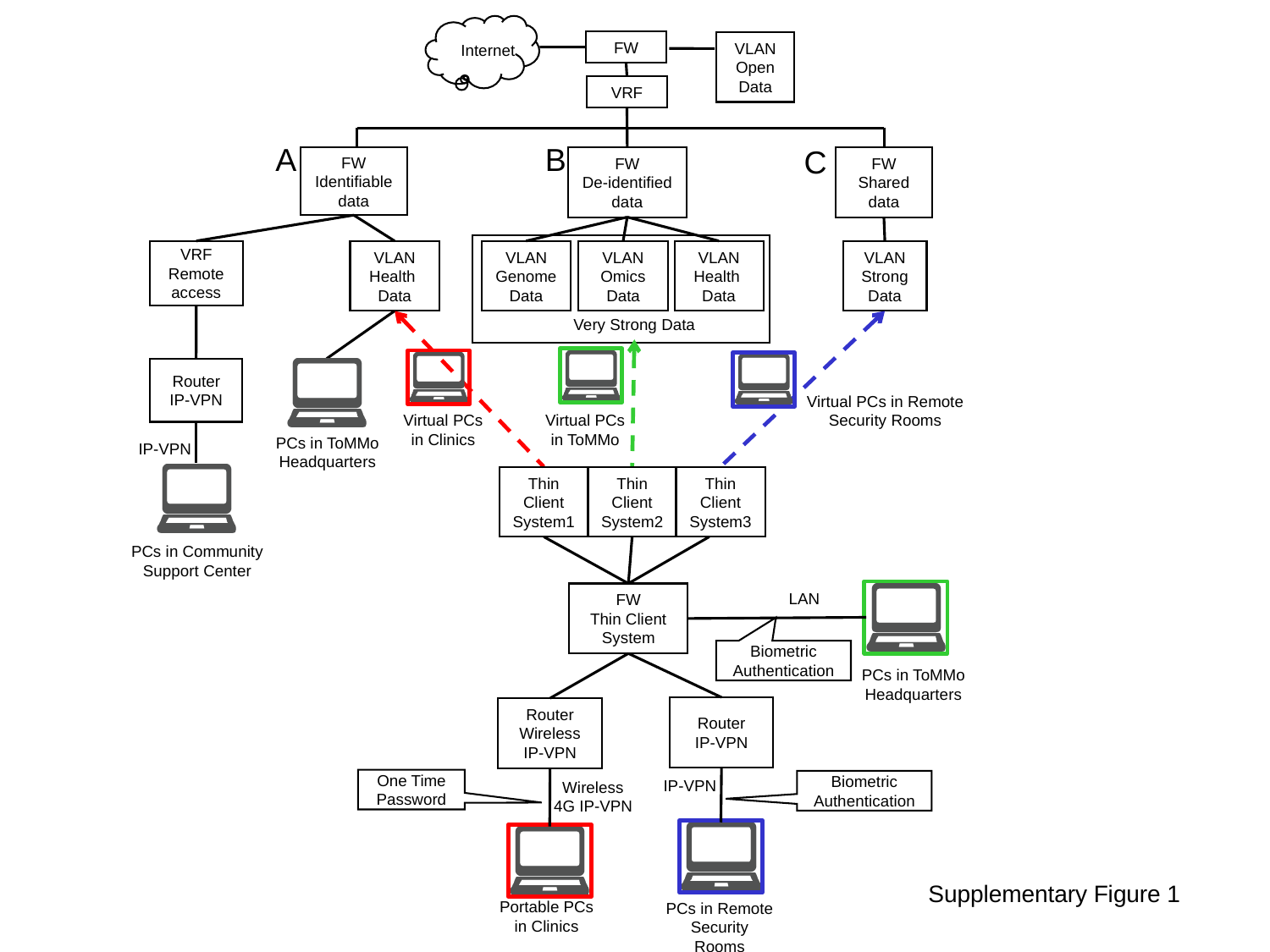

FW
VLAN
Open Data
Internet
VRF
A
B
C
FW
De-identified data
FW
Shared data
FW
Identifiable data
VRF
Remote access
VLAN
Health Data
VLAN
Genome Data
VLAN
Omics Data
VLAN
Health Data
VLAN
Strong Data
Very Strong Data
Router
IP-VPN
Virtual PCs in Remote Security Rooms
Virtual PCs in Clinics
Virtual PCs in ToMMo
PCs in ToMMo Headquarters
IP-VPN
Thin Client System1
Thin Client System2
Thin Client System3
PCs in Community Support Center
LAN
FW
Thin Client System
Biometric Authentication
PCs in ToMMo Headquarters
Router
IP-VPN
Router
Wireless IP-VPN
One Time Password
IP-VPN
Wireless 4G IP-VPN
Biometric Authentication
Supplementary Figure 1
Portable PCs in Clinics
PCs in Remote Security Rooms
