# Supplementary material for: Security controls in an integrated Biobank to protect privacy in data sharing: rationale and study design
Source: BMC Med Inform Decis Mak. 2017 Jul 6;17:100. doi: 10.1186/s12911-017-0494-5 (PMC5501115; doi:10.1186/s12911-017-0494-5)
Supplement: Supplementary file 3 — Working status of networks in the TMM biobank. The statistics of working status of the TMM networks is shown. The network configuration is shown in Additional file 2: Figure S1. (DOCX 12 kb) [file 12911_2017_494_MOESM3_ESM.docx]

**Additional file 3: Table S2: Working status of networks in the TMM biobank**

| **Subnetwork^1^** | **User^2^** | **Terminal^3^** | **Running time^4^** |
| --- | --- | --- | --- |
| Network for identifiable data | 299 | 197 | 4 years |
| Network for de-identifiable data | 168 | 78 | 4 years |
| Network for shared data | 8 | 11 | 1 years |

^1^Subnetworks that form an infrastructure of the TMM biobank, ^2^The number of users of the individual subnetworks, ^3^The number of terminals that connect to the individual subnetworks, ^4^Running time of the individual subnetworks. All the numbers were as of January 20, 2017.
